# Dose-dependent effect of silver nanoparticles (AgNPs) on fertility and survival of *Drosophila*: An *in-vivo* study

**DOI:** 10.1371/journal.pone.0178051

**Published:** 2017-05-24

**Authors:** Akanksha Raj, Prasanna Shah, Namita Agrawal

**Affiliations:** 1 Department of Zoology, University of Delhi, Delhi, India; 2 Acropolis Institute of Technology and Research, Indore, India; National Cheng Kung University, TAIWAN

## Abstract

Silver nanoparticles (AgNPs) containing consumer products have been proliferating in the market due to its unique antimicrobial property, however, lack of in-depth knowledge about their potential effect on human health in a longer run is of great concern. Therefore, we investigated dose-dependent *in vivo* effect of AgNPs using *Drosophila* as a model system. *Drosophila*, a genetically tractable organism with distinct developmental stages, short life cycle and significant homology with human serves as an ideal organism to study nanomaterial-mediated toxicity. Our studies suggest that ingestion of AgNPs in *Drosophila* during adult stage for short and long duration significantly affects egg laying capability along with impaired growth of ovary. Additionally, dietary intake of AgNPs from larval stage has more deleterious effects that result in reduced survival, longevity, ovary size and egg laying capability at a further lower dosage. Interestingly, the trans-generational effect of AgNPs was also observed without feeding progeny with AgNPs, thereby suggesting its impact from previous generation. Our results strongly imply that higher doses of AgNPs and its administration early during development is detrimental to the reproductive health and survival of *Drosophila* that follows in generations to come without feeding them to AgNPs.

## 1. Introduction

The outstanding feature that makes nanoparticles behave differently than their bulk counterparts is their relative size in the scale of nanometers. The altered property of nano-sized particles is attributed mainly to their highly increased surface area to volume ratio than their bulk counterparts [1]. In the past few years, owing to these extraordinary properties, nanoparticles have gained significant attention and became part of many consumer products [2]. Nanoparticles hold a great promise to improve the quality and efficiency of consumer goods. From ancient time, silver has been well acknowledged for its extraordinary bacteriocidal property making it part of food and medicine [3–7]. With the advancement and better understanding of nanotechnology, silver is being specifically engineered as silver nanoparticles (AgNPs) and used in a wide variety of consumer products [8] such as cosmetics [9–10], food packaging, wound dressing [11–13], biomedical devices [14–15], clothing, disinfectant products, textiles [16] and also for diagnostic and therapeutic applications [4, 17–18]. The exact mechanism by which AgNPs cause antimicrobial effect is not fully elucidated yet, however, it has been proposed that the interaction between AgNPs and bacterial cell results in generation of reactive oxygen species (ROS) and cell lysis [19–20]. AgNPs can access to the human body through direct skin contact or ingestion, and have the potential to subsequently propagate to the secondary target organs where they damage the cellular structures and DNA, causing tissue injury [4].

In addition, as AgNPs are extremely reactive, they themselves are considered a ROS generator. Most of the preliminary evidence from *in vitro* studies has proposed that AgNPs causes disruption of mitochondrial respiratory chain and thereby eliciting oxidative stress [21–28]. AgNPs are also reported to damage mammalian germ cells and have the potential to cause impairment of reproductive functions [29]. Limited number of *in vivo* studies have been performed which evidently show that nanoparticles represent a serious health threat [30–31] and may induce oxidative stress, apoptotic response [32–34] and expression of heat shock proteins [18]. A study in rat showed accumulation of AgNPs in different organs that can cause severe damage to these organs [35]. Additionally, AgNPs intake in *Drosophila* larvae can induce pigmentation defects, reduction in body size, loss of body weight and poor locomotor ability of adult flies [36–37]. All these reports on AgNPs strongly suggest that the negative impact of these nano sized particles on living organisms are of a serious concern, particularly if used in abundance.

Despite uncountable benefits of AgNPs, a systematic dose-dependent study to monitor impact of AgNPs exposure on human health is warranted. Therefore, with this mandate, we carried out *in vivo* dosage study of AgNPs to understand its effect on survival and fertility. Besides understanding dosage effect of AgNPs on adult parental population and progeny, a study of trans-generational effect is also very important to evaluate its long term impact. The experimental limitations and ethical restrictions involving *in-vivo* studies in higher mammalian system or human make trans-generational study challenging and therefore, *Drosophila* can serve as one of the most suitable model to address trans-generational effect of AgNPs. Using *Drosophila* as a model system, present study also aimed towards understanding systematic dose-dependent implications of ingestion of AgNPs on fertility and survival in few generations.

## 2. Materials and methods

### 2.1 Fly strain and culture

Wild-type *D*. *melanogaster* flies (Oregon-R) were raised on standard food containing maize flour, yeast, sugar, agar-agar and propionic acid at 25±1°C.

### 2.2 Characterization of AgNPs

AgNPs were purchased from Sun Innovations Corp., USA (Item# SN1101). Characterization of these commercially available AgNPs such as physical dimension, agglomeration state used in the present study has been reported previously [37]. However, to validate the quality and stability of the AgNPs used in our assessment, we characterized the particle size, size distribution, shape, composition, average crystallite size and stability of AgNPs using the measurements as follows: the average hydrodynamic diameter of monodispersed AgNPs was evaluated by DLS (Malvern Instrument Zetasizer Nano-ZS, Malvern, USA). The morphological measurement of AgNPs was performed by 200 keV Technai G2 UG20 TEM using a tungsten filament. Copper grid was used and the suspension of AgNPs was made in distilled water. The XRD measurement was carried out using Bruker D8 Advance X-ray diffractometer. The X-rays were produced using a sealed tube and the wavelength of X-ray was 0.154 nm (Cu K-alpha). The X-rays were detected using a fast counting detector-based on Silicon strip technology (Bruker Lynx Eye detector). The stability of AgNPs in deionized water was estimated using zeta potential measurement (Malvern Instrument Zetasizer Nano-ZS, Malvern, USA).

### 2.3 Administration of AgNPs in *Drosophila* food

A stock of 5% (w/v) AgNPs suspension in distilled water was sonicated (Q_SONICA_ Sonicators) at a pulse of 20 seconds, at an amplitude of 30% for 30 minutes to get a homogenous dispersion [37]. For analyzing the effect of different doses of AgNPs ingestion on fertility and survival, particles were sonicated in deionized water to make final doses of 5, 25, 50 and 250mg/L in partially cooled fly food. AgNPs supplemented food was stored in appropriate condition to avoid exposure to light.

**Fig 1:**
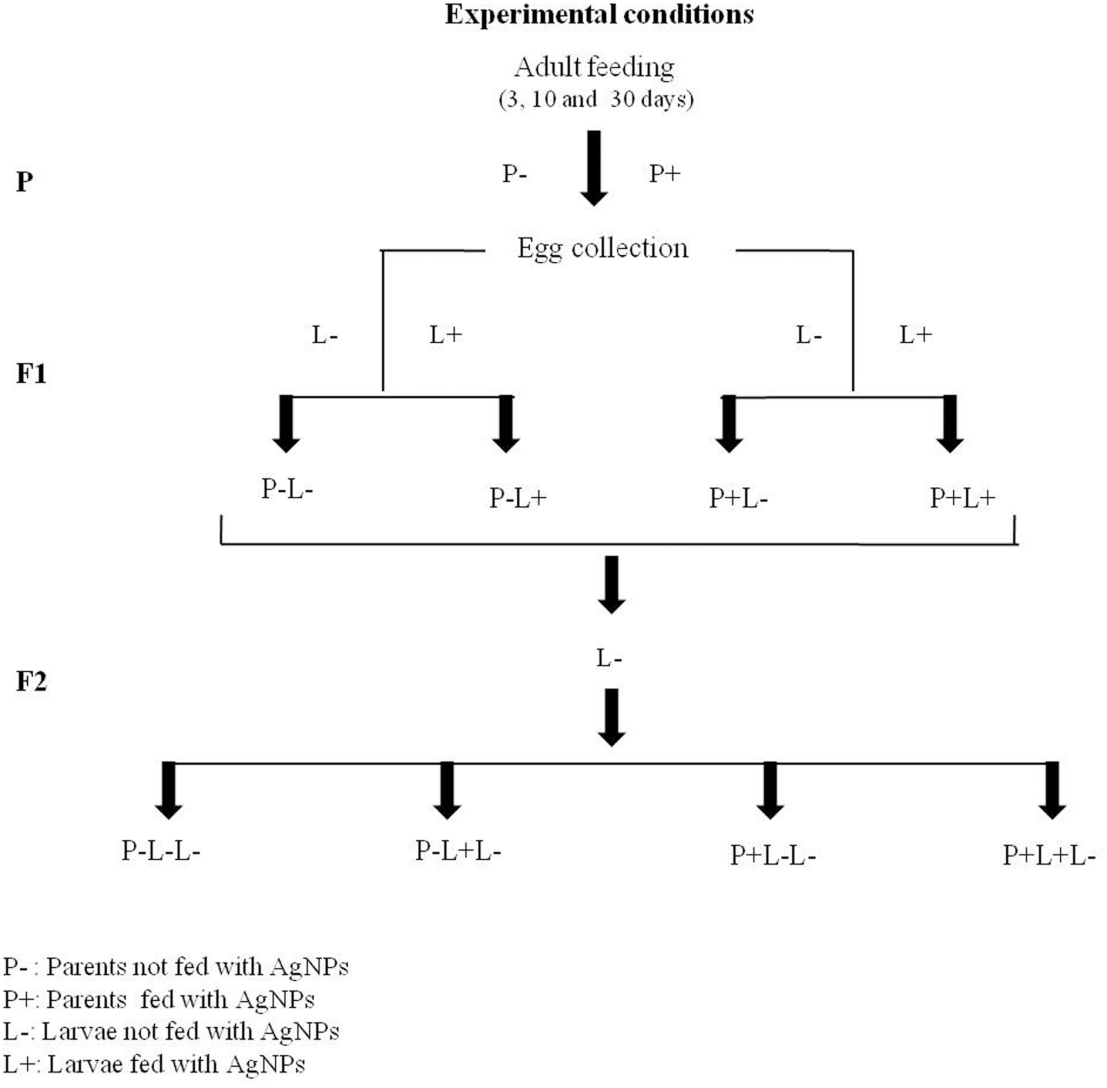
Schematic representation of AgNPs treatment conditions.

For parental adult feeding (P), three batches of freshly eclosed virgin female and male flies were fed separately on different doses of AgNPs-supplemented food for 3, 10 and 30 days. After feeding flies with different doses of AgNPs-supplemented food for respective time points, they were mated two days prior to egg laying. Eggs laid (F1) were transferred into the vials containing food without (control) and with AgNPs. Thus, four different treatments were given to F1 progeny i.e. referred as P-L- (Parents not fed; Larvae not fed), P-L+ (Parents not fed; Larvae fed), P+L- (Parents fed; Larvae not fed), P+L+ (Parents fed; Larvae fed) in subsequent text. A schematic illustration of these experimental conditions is shown in Fig 1.

Similarly, for trans-generational studies, the F1 adult progeny eclosing from the above treatments at different doses of AgNPs were allowed to mate and their eggs were transferred into the vials containing food without AgNPs. Thus, for F2 generation, the treatment conditions are referred as P-L-L- (Parents not fed; F1 Larvae not fed; F2 Larvae not fed), P-L+L- (Parents not fed; F1 Larvae fed; F2 Larvae not fed), P+L-L- (Parents fed; F1 Larvae not fed; F2 Larvae not fed), P+L+L- (Parents fed; F1 Larvae fed; F2 Larvae not fed) where F2 larvae were untreated. Here P-L- and P-L-L- represents control for F1 and F2 generation respectively.

Three sets of independent experiments were carried out for each assay. 75 eggs transferred in each food vial with at least 5 vials for each condition. So a total of 375 eggs for each condition were considered.

### 2.4 Analysis of egg laying capability

Female virgins and male flies were collected within 8 hours of eclosing, aged on different doses of AgNPs supplemented food followed by mating for 2 days on AgNPs food prior to monitoring their egg laying capability. Chambers housed with a set of 50 mated females in two replicates were allowed to lay eggs on standard fly food (without AgNPs) for four hours at 25±1°C. Number of eggs laid by females within four hours were counted to determine their rate of egg laying.

### 2.5 Assessment of *Drosophila* development and survival

Both control and AgNPs treated flies were allowed to lay eggs and 75 eggs from each condition were transferred in vials containing food supplemented without and with different doses of AgNPs. The larval hatch rate was determined by counting the number of 1^st^ instar larvae hatched within 24–28 hours after oviposition. Development upto pre-pupal and pupal stage was determined by counting the number of pupae formed out of 75 eggs transferred in food vials. Similarly, adult eclosion rate was determined by scoring the number of adult flies emerged from the pupae.

### 2.6 Life span assessment

For longevity of F1 flies, freshly eclosed non-mated female flies were collected in the group of 20 from each condition (P-L-/ P-L+/ P+L-/ P+L+). Flies were transferred into fresh food vial (without AgNPs) at an interval of 2 days and numbers of dead flies were recorded for 30 days. There were 4 replicates for each condition.

### 2.7 Statistics

Statistics was performed in IBM SPSS statistical package (version 22) for each experiment conducted. Statistical analysis was performed using analysis of variance (ANOVA) followed by Tukey’s post hoc test. The difference among treatment conditions were compared by Tukey—Kramer Minimum Significant Difference test (MSDα0.05) [38].

## 3. Results

### 3.1 Physico-chemical characterization of AgNPs

The AgNPs used for the present study (purchased from Sun Innovation Corp., USA) have been characterized and reported previously [37]. However, in the present study we have further confirmed its physico-chemical properties by using different characterization techniques.

**Fig 2:**
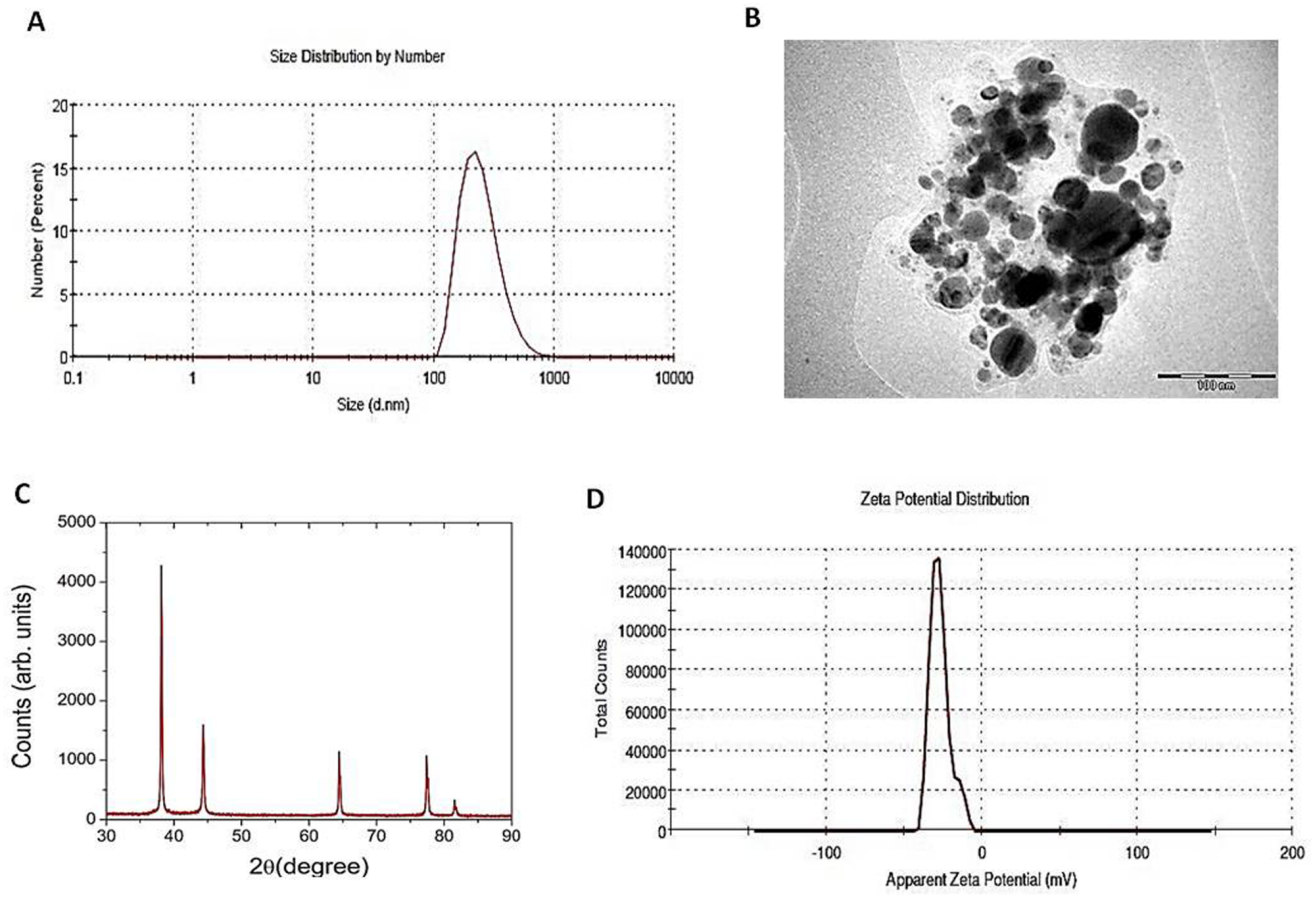
Characterization of AgNPs. **(A)** An average hydrodynamic size of mono-dispersed sample containing AgNPs is 252.9 nm by DLS measurements **(B)** TEM image of AgNP_S_ in suspension on Cu grid are in the range of 20–100 nm and spherical in shape. Majority of these particles fall in the size range of 20-50nm **(C)** X-ray diffraction measurements displayed the average crystallite size to be ~ 47 nm which is obtained by using Scherrer’s formula. **(D)** Zeta potential value of -27.4 mv displayed moderate stability of AgNPs.

The particle size was measured by DLS and the average hydrodynamic size of mono-dispersed sample containing AgNPs was estimated to be 252.9 nm (Fig 2A). Scattered light intensity from AgNPs was measured at 25°C for 60 min (Malvern Instrument Zetasizer Nano-ZS, Malvern, USA). Further, TEM measurement revealed that AgNPs are spherical in shape with a range of 20–100 nm (Fig 2B). AgNPs have been reported to display strong tendency to agglomerate [37] and we also observed the same in TEM images.

The typical X-ray diffraction pattern of the AgNPs is shown in (Fig 2C). The XRD data for AgNPs shows diffraction peaks at 2θ = 38.15, 44.34, 64.5, 77.45, 81.6 degrees which can be indexed to (111), (200), (220), (311), (222) planes of pure silver. XRD pattern confirms that the main composition of the nanoparticles is silver. The results of the curve fitting of the (111) peak gives a full width at half maximum (FWHM) value to be 0.2°. This included an instrumental broadening of 0.03°. The average grain size was found to be 47 nm, calculated by Scherrer’s formula (Fig 2C).

To evaluate the stability of the AgNPs in suspension, zeta potential measurement was done by Malvern Instrument Zetasizer Nano-ZS (Malvern, USA). Zeta potential value was −27.4 mv with poly dispersity index 0.5 that ensured moderate stability and agglomeration (Fig 2D).

### 3.2 Dose-dependent effect of AgNPs on egg laying capability of *Drosophila*

The effect of AgNPs on egg laying capability of adult flies was monitored by counting their eggs after feeding different doses of AgNPs supplemented food for short 3 and long 10, 30 days. A schematic representation of the experimental conditions is shown in Fig 1.Freshly eclosed female virgin flies were collected and fed with food supplemented without (control) and with different doses of AgNPs (5 mg/L, 25mg/L, 50mg/L and 250 mg/L) for 3, 10 and 30 days. Thereafter, two days prior to egg laying these females were mated with the age matched males that were also fed with AgNPs for respective days.

**Fig 3:**
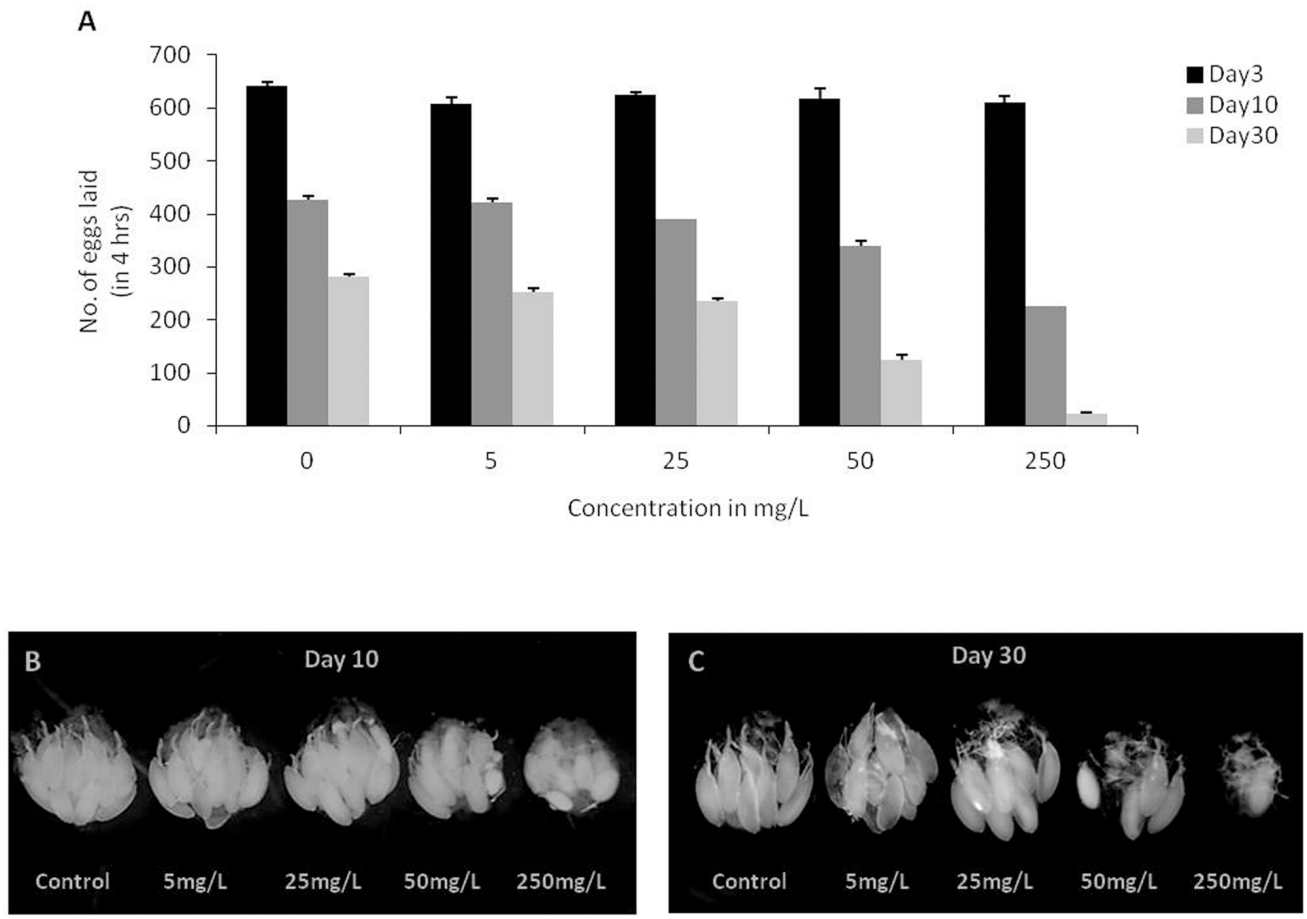
Dose dependent effect of AgNPs on egg laying and ovary development. **(A)** Egg laying capability of flies aged for 3, 10 and 30 days without (control) and with AgNPs at different doses (5mg/L, 25mg/L, 50mg/L and 250mg/L) **(B)** A notable difference in the size of ovary was observed in the flies aged on control and AgNPs supplemented food for 10 days and **(C)** 30 days in a dose-dependent manner. Significance was calculated by using an analysis of variance (ANOVA) followed by Tukey-Kramer post hoc test (MSD α_0.05_ = 116.87).

After feeding flies with different doses of AgNPs supplemented food for 3 days, eggs laid in 4 hours by the females were scored. We did not find any difference in egg laying capability of flies not fed (control) and fed to different doses of AgNPs (Fig 3A).

Further, egg laying capability of flies fed with different doses of AgNPs for 10 days was monitored. The egg laying capability was found to be decreased in a dose-dependent manner as compared to the females aged on food without AgNPs i.e. control (Fig 3A). However, below 50mg/L of AgNPs dosage, the egg laying capability of female flies had no effect and is comparable to that of control female flies.

To further understand the effect of prolonged ingestion of AgNPs on fertility of adult flies, a different batch of newly emerged flies was fed on AgNPs supplemented food for 30 days. It has been reported previously that the egg laying capability of wild-type female flies gets compromised with age [39–40].Similarly, we also observed reduction in egg laying capability of control flies with age. Interestingly, ingestion of higher doses of AgNPs for prolonged time period further pronounced this age related compromised egg laying capability of the treated flies (Fig 3A).

To monitor if the compromised egg laying capability of AgNPs fed 3 days, 10 days and 30 days old flies is due to its impact on gonadal development ovary of these adult flies was dissected. No difference in the ovary size was observed in the flies aged for 3 days on AgNPs diet, however, a notable difference in the size of ovary was seen in flies fed with higher doses of AgNPs for longer period i.e., upto 10 and 30 days (Fig 3B and 3C). Our results suggest that prolonged ingestion of AgNPs at higher doses, in particular at a dosage of 250mg/L impairs egg laying capability that could be due to retarded growth of ovary with the formation of just one or two ovariole.

Further, to understand if the reduced size of ovary and thereby compromised egg laying capability of the flies fed with higher dose of AgNPs is due to deposition of AgNPs within these tissues, we observed sections of ovary using TEM. However, we could not find any deposition of AgNPs inside the ovary. These observations indicate that reduction in ovary size and egg laying capability is the systemic side effect of AgNPs ingestion.

### 3.3 AgNPs ingestion impairs growth and longevity of F1 flies

**Fig 4:**
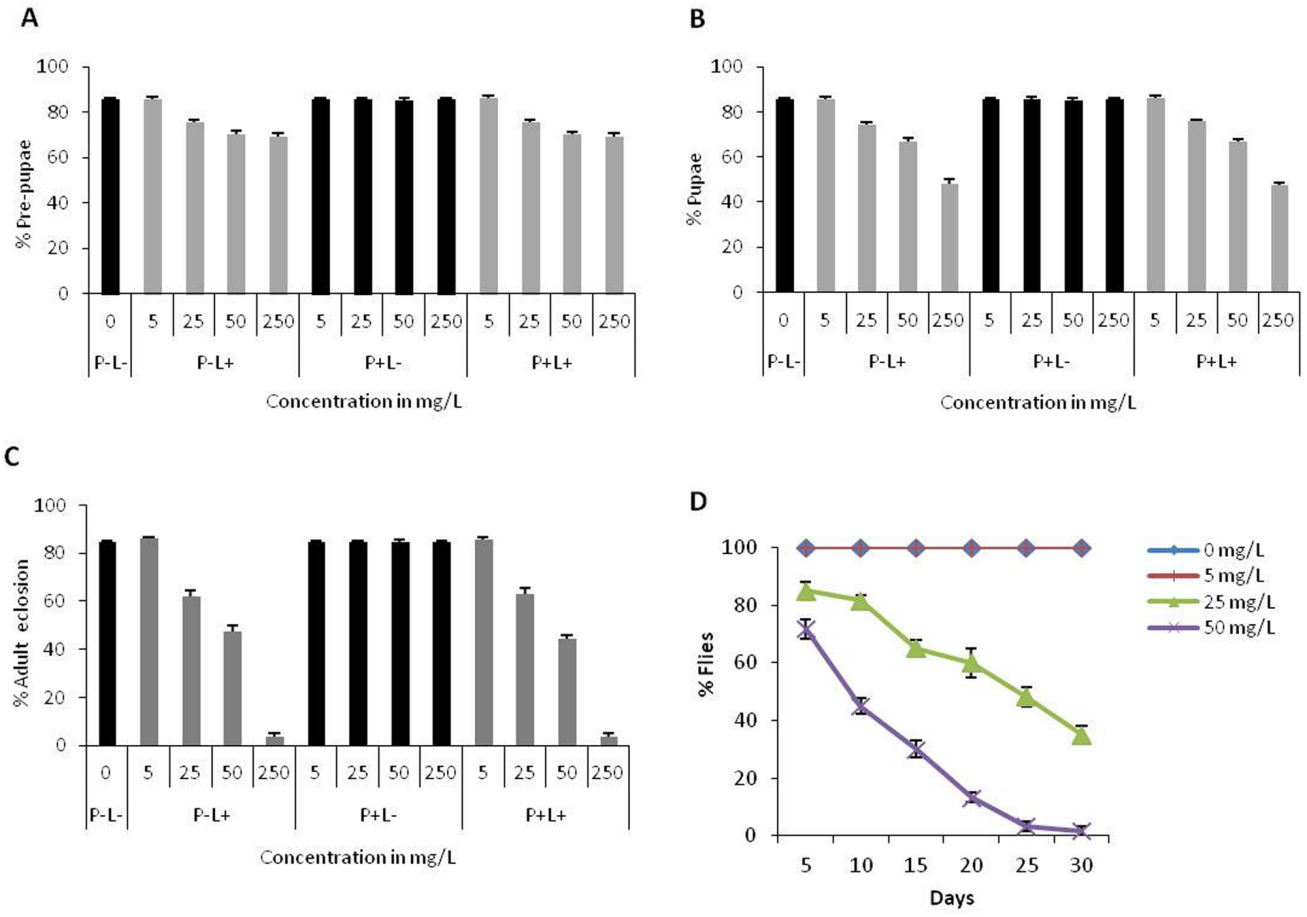
AgNPs ingestion during larval stage interferes with the growth and survival of F1 progeny. **(A)** Eggs reached to pre-pupal stage **(B)** pupa formation **(C)** adult eclosion rate and **(D)** longevity of F1 flies in P+L+ condition. Comparable results were observed for P-L+ and P+L+ conditions; P-L- represents control. Significance was calculated by using an analysis of variance (ANOVA) followed by Tukey-Kramer MSD post hoc test (MSD α_0.05_: Pre-pupal count = 14.62; Pupal count = 27.06; Adult eclosion = 27.06; Longevity = 35.34).

To find out development of the eggs laid by adult flies (P) fed with different doses of AgNPs for 3, 10 and 30 days, these eggs were transferred in food with various experimental conditions (P-L- i.e., both parents and larvae not fed; P+L- i.e., parents fed and larvae not fed; P-L+ i.e., parents not fed but larvae fed; P+L+ i.e., both parents and larvae fed). Following development of the eggs, pupal count and adult eclosion was monitored in different experimental conditions by calculating percentage of larvae that could reach the pre-pupal, pupal and adult stages. We found that AgNPs ingestion during the larval stage resulted in dose-dependent reduction in pre-pupae, pupae formation and adult eclosion (Fig 4A, 4B and 4C). We could get comparable results for pre-pupal and pupal formation from the parents fed with AgNPs for 3, 10 and 30 days; therefore, in the present manuscript the results are shown only for 10 days of adult feeding.

In addition to effects of AgNPs on different developmental stages, the adverse effect of larval AgNPs ingestion was also reflected on the longevity of F1 adult flies in a dose-dependent manner (Fig 4D). At 50mg/L of AgNPs, the number of flies that could survive was reduced to 50% within 10 days post eclosion; however, control flies were healthy and alive for 30 days. These results are very interesting as larval ingestion of AgNPs from 1^st^ instar onwards has impact on survival and longevity irrespective of feeding their parents with AgNPs.

### 3.4 Lower dosage of AgNPs ingestion early during development affects egg laying capability

**Fig 5:**
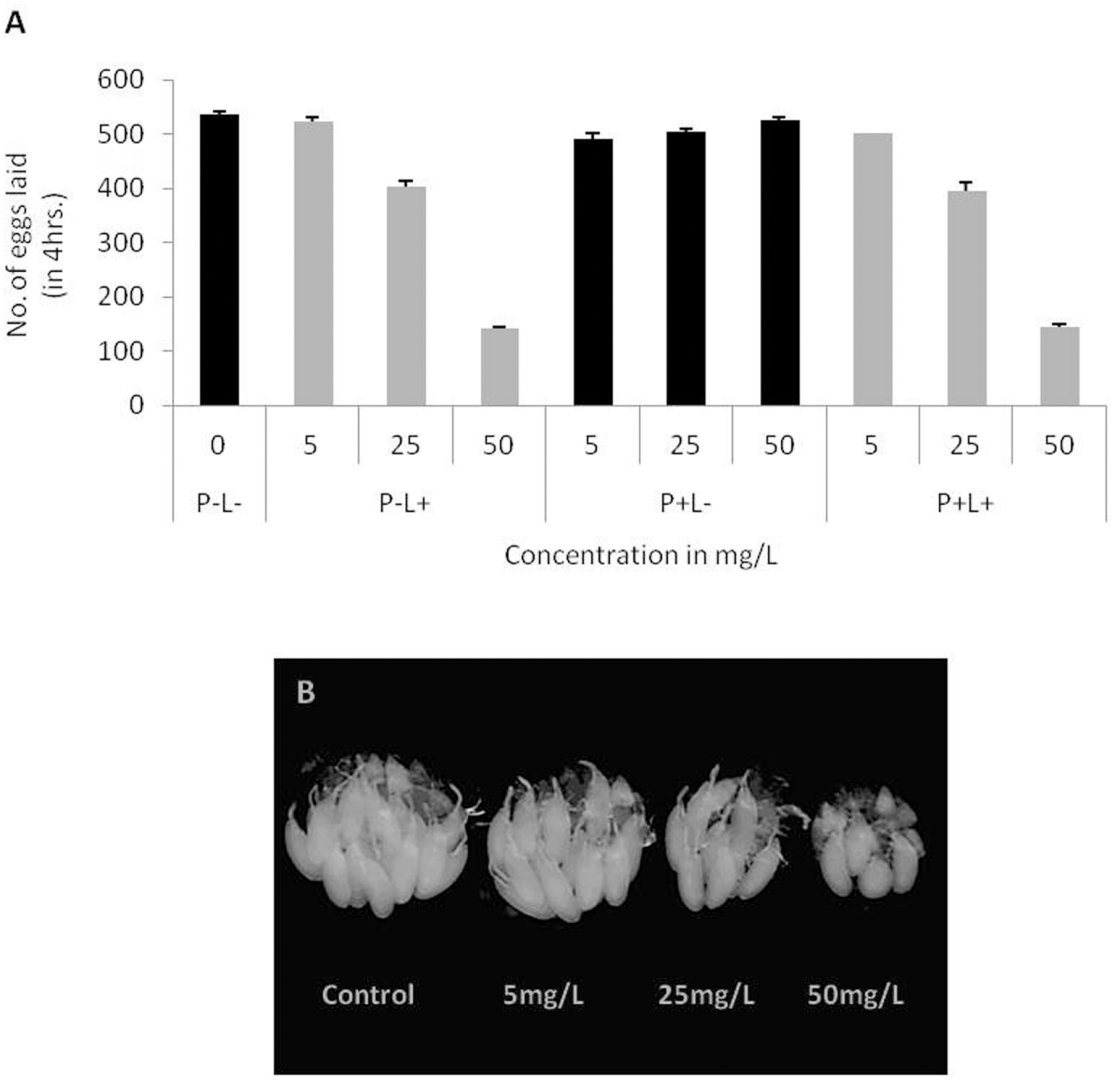
Impact of AgNPs ingestion at larval stage on egg laying and ovary development. **(A)** Egg laying capability of F1 female flies reduces substantially at higher dose. **(B)** Improper growth of ovary in P-L- (control) and P-L+ (at different doses) females. Statistical significance was calculated by using an analysis of variance (ANOVA) followed by Tukey Kramer MSD post hoc test (MSD α_0.05_ = 85.4).

The F1 adult eclosing from larvae fed with different doses of AgNPs displayed dose dependent decline in egg laying capability regardless of parents not fed (P-L+) or fed (P+L+) to AgNPs. It is interesting to observe that larval ingestion of AgNPs even at lower dose (25mg/L) could display compromised egg laying capability of F1 progeny. It is noteworthy that this dosage is safe for parental adults (Fig 5A). A significant decline in egg laying capability was observed at 50mg/L AgNPs. These results suggest that dietary intake of even lower dosage of AgNPs at early developmental stages has an impact as compared to the feeding during adult stage. We could also observe dose-dependent reduction in ovary size in the females eclosed from larvae fed with AgNPs at early larval stages (Fig 5B).

Dietary intake of AgNPs at larval stage also resulted in impaired oviposition capacity and development of ovary. However, we did not observe accumulation of AgNPs in ovary of these adults as well.

### 3.5 Trans-generational effect of AgNPs ingestion early during development

To evaluate if oral intake of AgNPs early during development can have trans-generational effect, we monitored growth and survival of F2 generation without feeding them with AgNPs. F1 adult flies eclosing out from (P-L-; P-L+; P+L- and P+L+ conditions) were fed with different doses of AgNPs (5mg/L, 25mg/L, 50mg/L and 250mg/L). Eggs from these flies were developed on normal food that is devoid of AgNPs to monitor trans-generational effect.

**Fig 6:**
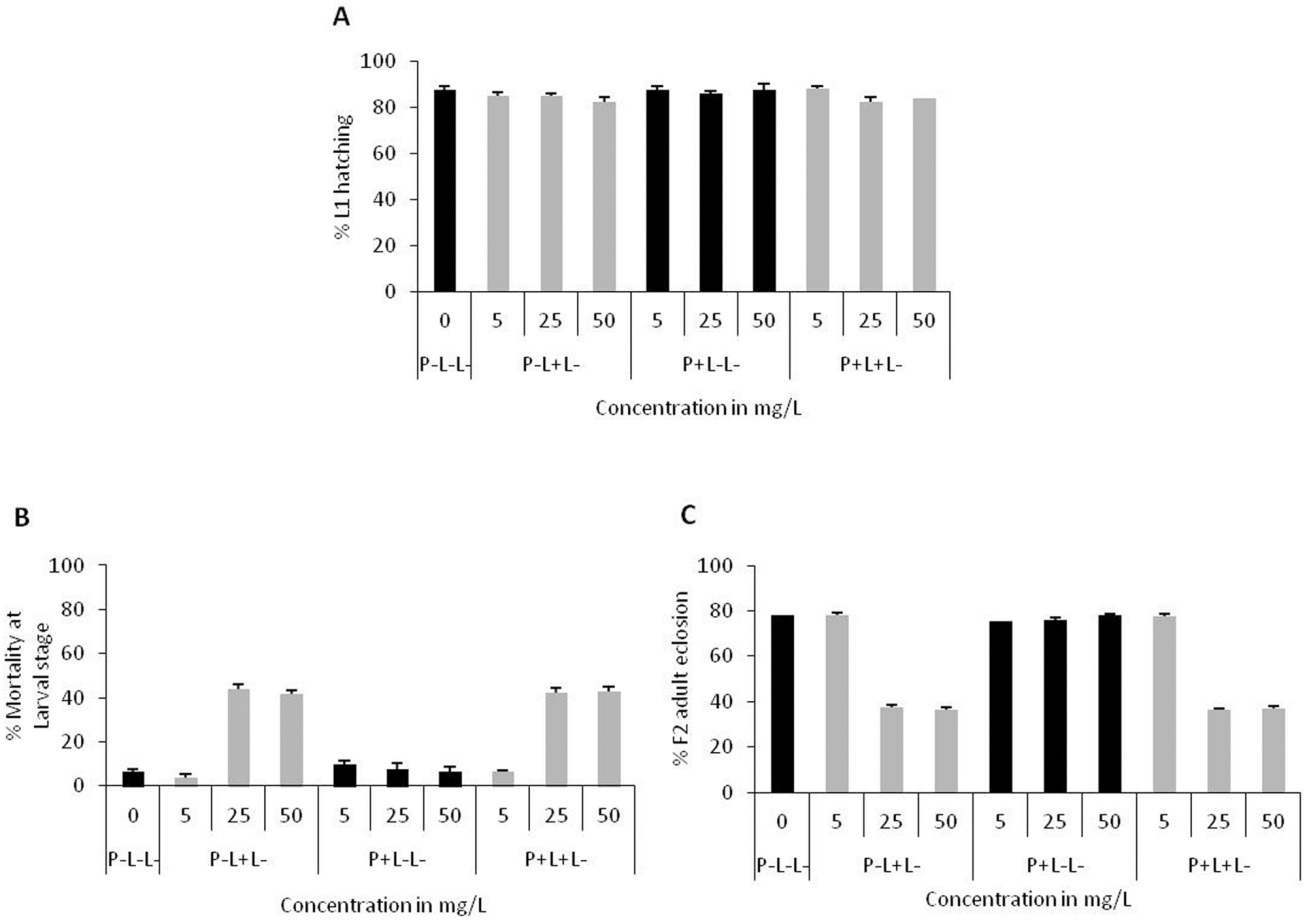
Trans-generational effect of AgNPs. **(A)** Hatching rate of first instar larvae of F2 generation in the absence of AgNPs. (**B**) Percentage mortality during 2^nd^ and 3^rd^ larval instars and (**C)** Percentage of F2 adults eclosed. Significance was calculated by analysis of variance (ANOVA) followed by Tukey-Kramer MSD post hoc test (MSD α_0.05_: Larval mortality = 27.6; F2 adult eclosion = 12.09).

We found significant reduction in survival of F2 progeny in the treatment conditions i.e., P-L+L- (Parents not fed (P-), F1 fed during early larval stage (L+), F2 not fed (L-) and P+L+L- (Parents fed (P+), F1 fed during early larval stage (L+), F2 not fed (L-).The percentage of eggs that hatched to 1^st^ instar larvae were comparable in all the treatment conditions i.e. P-L-L-; P-L+L-; P+L-L- and P+L+L-(Fig 6A). However, the percentage of 1^st^ instar larvae that reached to pre-pupal stage declined significantly in two conditions i.e. P-L+L- and P+L+L-, suggesting that maximum mortality in F2 generation occurs between 2^nd^ to 3^rd^ larval instar only when early stage larvae of F1 generation were fed with AgNPs (Fig 6B). As a consequence of larval mortality, adult emergence was also reduced significantly (Fig 6C).

The mortality rate during larval stages in F2 generation followed dose-dependent impact of AgNPs as observed in F1 generation parents. These results strongly suggest that if parents are fed with higher doses of AgNPs early during development, the effect gets carried over to the next generation.

## 4. Discussion

With the advent of newer applications of AgNPs, their frequent exposure to human through consumer goods without detailed understanding of their impact remains a major concern. In order to address this emerging consensus about the putative health risks associated with their long term application, a detailed and systematic *in vivo* study was conducted using *Drosophila melanogaster* as a model system. In view of the short lifespan and significant concordance with human, *Drosophila* is a very convenient model for trans-generational *in vivo* studies.

The commercially manufactured AgNPs (35nm) from Sun Innovations Corp., USA was used to investigate the dosage effect in the present study. As a mandate to monitor nanotoxicity, physico-chemical characterization of these commercially purchased AgNPs was carried out. TEM and DLS studies demonstrated that AgNPs used in present study are spherical in shape with a size distribution range of 20-100nm and hydrodynamic diameter of 252 nm. Further, the zeta potential measurement of dispersed AgNPs in deionized water indicated that these nanoparticles are moderately stable. All these physico-chemical measurements strengthened the present study to evaluate impact of AgNPs ingestion in *Drosophila*.

To elucidate the dosage effect of intake of AgNPs on developmental stages and time duration, first instar larvae and adults for short and long duration were administered with AgNPs supplemented food. Our results strongly suggest that adults are more resistant to AgNPs ingestion as it has no effect on their survival. However, dietary intake of AgNPs in larvae affects the rate of adult emergence and lifespan of their progeny in a dose-dependent manner. The most alarming results were observed from intake of AgNPs during early larval stage that is being carried forward to the next generation. A significant reduction was observed in the percentage of larvae reaching adulthood even when reared in the absence of AgNPs supplemented food, suggesting trans-generational effect of AgNPs.

Besides survival, the consequence of AgNPs ingestion on fertility was monitored by analyzing egg laying capability and growth of the ovary of not fed and fed flies. Prolonged ingestion of AgNPs-supplemented food in adult flies reduced their rate of oviposition in a dose-dependent manner Additionally, AgNPs also interferes with the attainment of proper growth of ovary in female flies at higher doses. It is interesting to observe that size of the ovary and egg laying capability of F1 adult flies gets significantly affected when reared on diet at further lower AgNPs dose. However, TEM analysis of sections of ovary from these AgNPs fed flies did not show any deposition of AgNPs. These results suggest that the reduced reproductive ability of AgNPs fed flies could be systemic side effect of AgNPs ingestion.

Oogenesis in *Drosophila* has been previously reported to be highly sensitive to the nutritional alterations. Nutrition is known to play a very important role in development of ovary and determining the female egg laying behavior [41]. The observed ovarian defect and impaired rate of egg production of P and F1 flies by rearing them on AgNPs-supplemented food can be well attributed to nutritional deficiency (Raj et al; unpublished data). Additionally, the role of insulin signaling and ecdysone response pathway in regulating the ovary development has been reported [42–44]. There is a possibility that ingestion of AgNPs may be interfering with these pathways as well that result in formation of small ovary lacking oocyte. As a result of abnormal ovary development, an adverse impact on the reproductive fitness of the female flies causes impaired egg laying and further development [45–46].

From our findings, it can be concluded that effect of AgNPs ingestion is dose and developmental stage specific. It is interesting to observe that impact of AgNPs ingestion during early larval stage is being retained in the next generation. The possible reason for lethality caused by the higher dose of AgNPs ingestion during early larval stage could be due to generation of ROS [26, 32–34]. The mechanisms through which intake of AgNPs at F1 larval stage may cause trans-generational effect could be multifactorial. The observed phenomena of trans-generational effect could be due to impaired germ cells [47–50]. Additionally, ingestion of AgNPs in larvae is reported to have genotoxic potential in *Drosophila melanogaster* [51–52], which could also be one of the mechanisms behind the trans-generation effect.

We finally conclude that AgNPs being one of the commonly used nanoparticles in consumer products and therefore, it is necessary to evaluate safe dosage to minimize human health related issues.
